# THE ROLE OF EDUCATORS AND EMPLOYERS IN REDUCING (OR PERPETUATING) AGEISM

**DOI:** 10.1093/geroni/igz038.183

**Published:** 2019-11-08

**Authors:** Tina M Kruger

**Affiliations:** Indiana State University, Terre Haute, Indiana, United States

## Abstract

As the population ages, increasing numbers of people are at risk of being harmed by ageism found in interpersonal interactions, medical settings, employment opportunities, and public policies. The way older people are talked to and about can facilitate the inclusion or exclusion/dismissal of the older population. Gerontology educators are well-positioned to combat ageism by discussing ageist beliefs with students and by teaching about stereotype development, aging stigma, and inclusive language and interactions. Aging services providers can address ageism by hiring the candidates most qualified to engage with older adults in a non-ageist manner. We explore these ideas in this symposium. First, information from the Gerontological Literacy Network’s Sketches study regarding college students’ (mis)perceptions of aging will be presented. Second, the Ageism First Aid online training modules, designed to reduce ageism, will be introduced. Third, the Disrupt Aging Classroom practice model, created by AARP CT, and Borrow My Glasses, used to transform attitudes about aging among college students, will be shared. Fourth, the disjoint between aging services job descriptions and how those who study aging/gerontology search for jobs will be reviewed. Finally, data from the GELS project, regarding where gerontology program graduates have gained employment, will be presented. Efforts to educate all college students and aging services providers about appropriate language and interaction styles, combined with modifying aging services employers’ efforts to identify the most qualified candidates to hire, can ultimately reduce ageism and enhance quality of life for the fastest growing segment of the population.

